# Evaluation of proposed cranial and maxillary bone alteration parameters in persons affected by Hansen’s disease

**DOI:** 10.1371/journal.pntd.0009694

**Published:** 2021-08-25

**Authors:** Rachel Bertolani do Espírito Santo, Dâmaris Versiani Caldeira Gonçalves, Rachel Azevedo Serafim, Rafael Maffei Loureiro, Daniel Vaccaro Sumi, Ricardo Andrade Fernandes de Mello, Simon M. Collin, Patrícia Deps

**Affiliations:** 1 Department of Social Medicine, Postgraduate Programme in Infectious Diseases, Federal University of Espírito Santo, Vitória-ES, Brazil; 2 Department of Internal Medicine, Federal University of Espírito Santo, Vitória-ES, Brazil; 3 Imaging Department, Hospital Israelita Albert Einstein, São Paulo, Brazil; 4 National Infection Service, Public Health England, London, United Kingdom; Yale University School of Medicine, UNITED STATES

Most of the 200,000 new cases of Hansen’s disease (HD, leprosy) diagnosed each year occur in India, Indonesia and Brazil [1]. The impact of HD on individual affected persons and the overall burden on health systems is amplified by delayed diagnosis, which increases the risk of patients presenting with HD-related impairments and disabilities [2,3]. These can include bone alterations, particularly in the hands, feet and face [4], leading to secondary complications [5], long-term disability and diminished quality-of-life [6–8], and contributing to stigmatization of persons affected by HD.

The degree of bone alteration correlates with the form of HD, ranging from limited or undetectable changes in the tuberculoid form to severe changes at the ‘lepromatous’ pole [9]. Although severe bone changes as a consequence of HD are less prevalent than in the pre-treatment era and population incidence of new cases presenting with disabilities has declined in many countries [10,11], disabilities related to Hansen’s disease in the age of modern medicine are not uncommon [12,13], affecting a relatively constant or increasing proportion of new cases [10,14]. Endemic and non-endemic countries also have elderly former and current HD patients who are affected by HD-related disabilities [15,16].

Rhino-maxillofacial changes caused by *Mycobacterium leprae* infection of the nasal passages and the palate include collapse of the bridge of the nose, resorption of the central part of the maxilla, inflammation of the floor and walls of the nasal cavity and hard palate and, ultimately, perforation of the palate [17]. A set of 7 maxillofacial bone alteration criteria define a ‘rhinomaxillary syndrome’ (RMS) [18]. Externally, RMS manifests in changes to the facial profile, including: 1) saddle nose, characterized by loss of nasal dorsal height and shortened length of nose, due to cartilaginous and/or bone collapse; 2) concave middle-third of the face with sunken (retracted) nose, caused by erosion of the zygomatic process and enlargement and loss of the pyriform shape of the nasal aperture; 3) reduced maxillary projection (maxillary retrognathia/reduced ANS); 4) inverted upper lip because of reduced maxillary height [19].

Maxillofacial bone changes assessed *in vivo* by computed tomography (CT) imaging have been described in two studies [20,21]. The first, by Kasai *et al*., was a study of 10 former HD patients (age 68–97 years) residing in a National Sanatorium in Japan and 5 adults (age 36–91 years) with no history of HD. The authors reported that 4/10 participants had substantial (severe) maxillary changes and saddle nose whilst 3/10 had no bone alterations. Kasai *et al*. calculated two measures: a ratio of maxillary/cranial anterior-posterior length (M_A-P_/C_A-P_) and a ‘maxillary defect’ measure (calculated by subtracting the observed anterior-posterior maxillary length from the observed cranial length multiplied by the mean M_A-P_/C_A-P_ ratio in the non-HD group, with negative values indicating apparent defective length).

Kasai *et al*. found that the M_A-P_/C_A-P_ ratio appeared to be lower in cases (mean 0.52 ± SD 0.07) than in controls (mean 0.57 ± SD 0.02) and was especially low in the 3 of the 4 cases with severe alterations. On this basis, Kasai *et al*. suggested that the M_A-P_/C_A-P_ ratio could serve as a universal parameter to evaluate maxillofacial bone alterations among individuals with different cranial sizes. They also proposed that the maxillary defect value could be used to estimate the size of an implant, autologous bone or prosthesis for reconstruction of the deformed maxilla.

We conducted the second study, based on CT imaging of 16 former HD patients (age 60–89 years) resident at Pedro Fontes Hospital, Espírito Santo, Brazil. We reported that 4/16 former patients met the criteria for RMS, and we categorized a further 4 participants as having ‘partial RMS’ [21].

We have since obtained measurement data to calculate the M_A-P_/C_A-P_ ratio and maxillary defect (**Figs 1 and 2**) in the 16 former HD patients in our original study, in 21 current HD patients (age 33–77 years) and in 37 non-HD controls (matched by age and sex to the 37 former and current HD patients) (**Table 1**). We compared the measurements reported by Kasai *et al*. between all cases (current and former patients) and non-HD controls (**Table 1**), between current and former HD patients (**Table 1**), and between former patients with and without full or partial RMS (**Table 2**).

**Fig 1:**
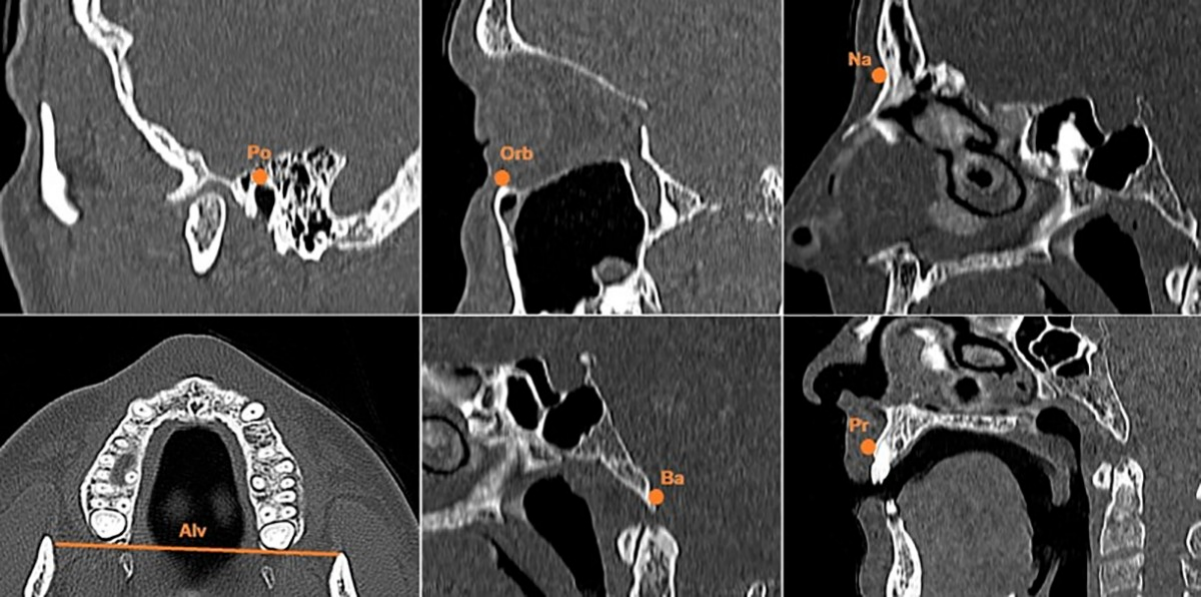
Standardized cranium reference points. Po = *porion; Orb = orbitale; Na = nasion; Alv = alveolon; Pr = prosthion; Ba = basion*.

**Fig 2:**
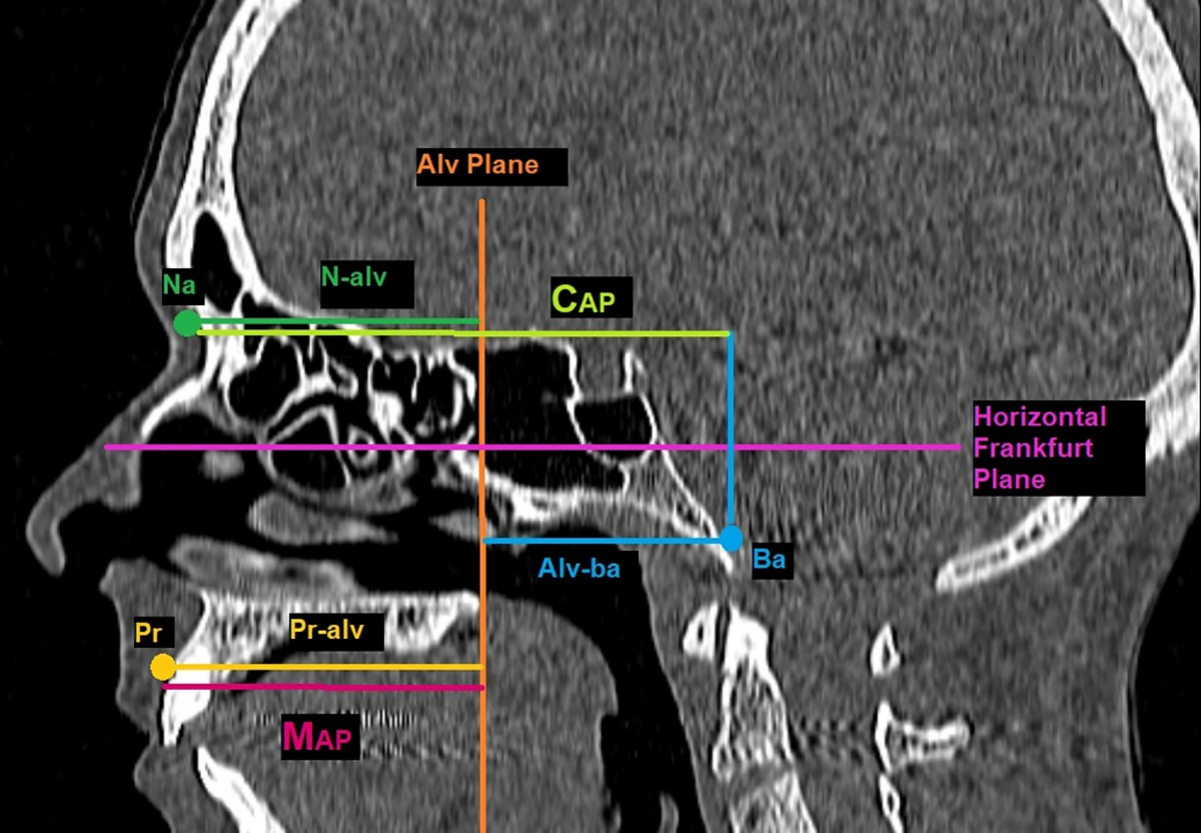
Standardized anteroposterior measurements of the cranium and the maxilla represented in the median sagittal plane. *Na* = *nasion*; *Pr* = *prosthion*; *Ba* = *basion*; N-Alv = distance between Alveolar (Alv) plane and *nasion;* Pr-Alv = distance between *prosthion* and Alv plane; Alv-Ba = distance between *basion* and Alv plane; C_AP_ = cranium anterio-posterior length = N-Alv + Alv-Ba. M_AP_ = maxilla anterio-posterior length = Pr-Alv.

**Table 1:** Demographic and cranial characteristics of current and former Hansen’s disease (HD) patients and non-HD controls.

	Non-HD control (n = 37)	Case (current or former HD patient) (n = 37)	P-value (cases *vs*. controls)^†^	Current HD patient (n = 21)	Former HD patient (n = 16)	P-value (former *vs*. current HD patients)^†^
Sex (male)	18 (48.7%)	18 (48.7%)	1.00	12 (57.1%)	6 (37.5%)	0.24
Age (years)	62 (57–70)	62 (58–70)	1.00	58 (47–60)	73 (66–78)	<0.001
n-alv distance (mm)	46.3 (44.3–48.4)	44.9 (42.0–47.2)	0.07	45.1 (42.0–47.2)	44.2 (41.9–46.8)	0.54
mean difference^‡^ (mm)	-1.35 (-2.92, 0.22)		0.09	-0.33 (-3.89, 3.23)		0.85
alv-ba distance (mm)	42.5 (38.2–44.4)	41.8 (39.1–44.1)	0.82	41.8 (39.1, 44.6)	41.7 (39.8–43.8)	0.67
mean difference^‡^ (mm)	0.17 (-1.57, 1.90)		0.85	-0.16 (-3.46, 3.13)		0.92
Cranial anterior-posterior length (C_A-P_)^¶^ (mm)	88.3 (84.1–91.0)	86.2 (84.2–90.2)	0.31	86.2 (83.4–91.3)	86.3 (84.3–88.4)	0.56
mean difference^‡^ (mm)	-1.18 (-3.18, 0.81)		0.24	-0.49 (-4.84, 3.86)		0.82
Maxilla anterior-posterior length (M_A-P_) (mm)	49.8 (47.3–53.2)	49.5 (47.1–53.4)	0.98	51.0 (47.1–54.8)	49.0 (46.7–51.0)	0.11
	-0.13 (-1.99, 1.73)		0.89	-0.24 (-3.99, 3.51)		0.90
M_A-P_/C_A-P_ ratio	0.57 (0.54–0.59)	0.58 (0.55–0.61)	0.43	0.58 (0.56–0.61)	0.57 (0.54–0.59)	0.37
mean difference^‡^ (mm)	0.007 (-0.013, 0.028)		0.49	-0.001 (-0.042, 0.040)		0.97
Maxillary defect (mm)^#^	-0.11 (-2.48–2.02)	-0.93 (-3.61–1.95)	0.48	-1.44 (-3.76–0.90)	-0.49 (-1.81–2.82)	0.37
mean difference^‡^ (mm)	-0.54 (-2.36, 1.27)		0.55	-0.04 (-3.63, 3.55)		0.98

^†^ Value shown are frequency (%), median (IQR) or mean difference (95% CI); p-value from Chi-squared test for categorical variables (Fisher’s exact if n≤5), Kruskal-Wallis test for continuous variables, t-statistic for linear regression mean difference

^‡^ Mean difference from linear regression adjusted for age and sex

^¶^ C_A-P_ = n-alv distance + alv-ba distance

^#^ Maxillary defect = (C_A-P_ x 0.567)—M_A-P_ where 0.567 = mean M_A-P_/C_A-P_ ratio in non-HD controls

**Table 2:** Demographic and cranial characteristics of former Hansen’s disease (HD) patients with and without rhinomaxillary syndrome (RMS).

	Former HD patient without RMS (n = 8)	Former HD patient with RMS (n = 8)	P-value^†^
Sex (male)	2 (25.0%)	4 (50.0%)	0.61
Age (years)	69 (66–78)	77 (66–78)	0.71
n-alv distance (mm)	44.9 (42.6–46.8)	43.8 (41.8–46.2)	0.53
mean difference^‡^ (mm)	-0.90 (-5.24, 3.44)		0.66
alv-ba distance (mm)	41.5 (39.8–45.3)	41.7 (39.0–43.6)	0.83
mean difference^‡^ (mm)	-2.24 (-6.48, 2.00)		0.27
Cranial anterior-posterior length (C_A-P_)^¶^ (mm)	86.5 (84.3–89.3)	86.1 (82.2–87.4)	0.60
mean difference^‡^ (mm)	-3.14 (-8.16, 1.88)		0.20
Maxilla anterior-posterior length (M_A-P_) (mm)	48.2 (44.1–51.0)	49.3 (48.4–51.4)	0.25
	1.42 (-2.24, 5.07)		0.42
M_A-P_/C_A-P_ ratio	0.55 (0.52–0.58)	0.59 (0.57–0.62)	0.05
mean difference^‡^ (mm)	0.041 (0.003, 0.079)		0.04
Maxillary defect (mm)^#^	1.85 (-1.02–3.74)	-1.45 (-3.63–0.04)	0.06
mean difference^‡^ (mm)	-3.20 (-6.45, 0.06)		0.05

^†^ Value shown are frequency (%), median (IQR) or mean difference (95% CI); p-value from Chi-squared test for categorical variables (Fisher’s exact if n≤5), Kruskal-Wallis test for continuous variables, t-statistic for linear regression mean difference

^‡^ Mean difference from linear regression adjusted for age and sex

^¶^ C_A-P_ = n-alv distance + alv-ba distance

^#^ Maxillary defect = (C_A-P_ x 0.567)—M_A-P_ where 0.567 = mean M_A-P_/C_A-P_ ratio in non-HD controls

We found no difference between any of the groups in the mean M_A-P_/C_A-P_ ratio or in any of the measurements used to calculate the ratio (**Table 1**). One component of the cranial anterior-posterior length tended to be shorter in cases than controls, but this difference was not supported by statistical evidence (p = 0.09, linear regression adjusted for age and sex). Among former patients, the M_A-P_/C_A-P_ ratio was higher in those with full or partial RMS (p = 0.04, linear regression adjusted for age and sex), but there were no differences in the components of the ratio (**Table 2**).

The mean M_A-P_/C_A-P_ ratio in our control group (0.567 ± SD 0.047) was the same as reported by Kasai et al. in their control group (0.57 ± SD 0.02), albeit with more variation in our controls. Median or mean maxillary defect did not differ between patients and controls or between current and former HD patients, but former HD patients with RMS had a larger defect (p = 0.05, linear regression adjusted for age and sex) than former HD patients without RMS. This finding is consistent with Kasai *et al*., who found large defects in 3/4 severely-affected patients, although absolute defect values for our 16 former HD patients (median -0.49mm, range -4.57mm to 6.17mm) were less substantial than in the data reported by Kasai *et al*. for their 10 patients (median -3.11mm, range -17.94 to 2.47mm).

We conducted a statistical test of the results reported by Kasai *et al*. and found insufficient evidence for the apparent difference in mean M_A-P_/C_A-P_ ratios between patients and controls (p = 0.15, Student’s t-test). We also fitted their data to a model adjusted for age and sex and found no differences between patients and controls in M_A-P_/C_A-P_ ratio (p = 0.16), its component measurements (M_A-P_ p = 0.48, C_A-P_ p = 0.31) or maxillary defect (p = 0.18).

We have therefore mostly replicated null findings, with the exception of some evidence for a higher M_A-P_/C_A-P_ ratio in former HD patients who had RMS (counter to Kasai *et al*.’s hypothesis that the ratio would be lower in more severely affected persons). We did confirm that the ‘maxillary defect’ measurement indicated persons with more substantial maxillary bone alterations, but whether this measure is of utility in planning corrective surgery is uncertain given that absolute differences were small and found in a group of elderly former HD patients in whom the risks of surgical intervention might outweigh any potential benefits.

The main limitation to our and Kasai *et al*.’s studies is that both were limited by small sample sizes. Definitive answers as to which radiological measurements might be most reliably associated with cranial and maxillary bone alterations caused by HD requires a larger study, ideally involving persons affected by HD from the main endemic countries. Although we controlled for age and sex, confounding by processes related to ageing and disease could affect our results.

Ultimately, future studies must aim to provide benefit to persons affected by HD by improving our understanding of how HD-related bone alterations progress before, during and after treatment, and by suggesting improvements to clinical practice. The latter could include enhanced assessment and follow-up to prevent secondary complications. In settings where provision of radiological services is resource-constrained, approaches based on otorhinolaryngological evaluation and examination of facial profile changes may suffice [19]. However, the increasing availability of CT scanning even in endemic countries means that these approaches can be supplemented, where cranial bone changes are suspected, by radiological imaging as a component of specialist care for persons affected by HD.

## Ethics statement

The studies from which data were obtained were approved by the Research Ethics Committee of the Health Sciences Center of the Federal University of Espírito Santo (1.101.787 on 10th June 2015 and 4.248.419 on 31st August 2020). Formal written informed consent was obtained from all participants.
